# Long-term outcomes following thrombolysis of arteriovenous grafts

**DOI:** 10.1177/11297298211027470

**Published:** 2021-11-19

**Authors:** Ben Li, Monica Abdelmasih, Naomi Eisenberg, Charmaine Lok, Graham Roche-Nagle

**Affiliations:** 1 Division of Vascular Surgery, Department of Surgery, Toronto General Hospital, University Health Network, University of Toronto, Toronto, ON, Canada; 2 Division of Nephrology, Department of Medicine, Toronto General Hospital, University Health Network, University of Toronto, Toronto, ON, Canada

**Keywords:** Arteriovenous graft, thrombolysis, patency, long-term, outcomes

## Abstract

**Background:**

Thrombolysis for arteriovenous grafts (AVG) yields high technical success rates, however, long-term outcomes are unclear. We conducted a multicenter retrospective cohort study to analyze 5-year patency rates following AVG thrombolysis.

**Methods:**

All patients who underwent AVG thrombolysis between 2005 and 2015 at three academic hospitals were included. Prospectively maintained institutional nephrology and radiology databases were used to record demographic, clinical, and AVG characteristics. The primary outcome was primary patency, defined as AVG access survival without re-intervention including angioplasty ± stent with/without re-thrombolysis. Secondary outcomes were assisted primary patency and cumulative patency, defined as AVG access survival until re-thrombosis requiring re-thrombolysis or abandonment, respectively. Technical success was defined as restoration of flow with <30% residual stenosis. Patients were followed until 2017. Patency rates were assessed using Kaplan–Meier survival analysis and Cox proportional hazards were calculated to determine associations between covariates and patency loss.

**Results:**

Seventy-four patients underwent AVG thrombolysis during the study period with a median follow-up period of 21.4 (IQR 8.3–42.8) months. The average age was 58.6 years with a high rate of comorbidities, including hypertension (82.4%) and diabetes (54.1%). Thrombolysis technical success was 96%. There were 147 re-interventions in 46 patients, of which 98 were re-thrombolysis (mean re-intervention rate of 1.27/patient/year). Primary patency at 1, 3, and 5 years were 43.2%, 20.2%, and 7.7%. Assisted primary patency at 1, 3, and 5 years were 47.5%, 20.2%, and 7.7%. Cumulative patency at 1, 3, and 5 years were 75.0%, 38.8%, and 22.6%. Cox proportional hazards analysis demonstrated no associations between demographic, clinical, and procedural characteristics and patency rates.

**Conclusions:**

Despite a high technical success rate, thrombolysis for AVG dysfunction is associated with poor long-term patency. Future studies are needed to determine risk factors for re-thrombosis to identify patients who will benefit from AVG thrombolysis in the long-term.

## Background

Arteriovenous grafts (AVG) provide an alternative route for hemodialysis in patients who are anatomically unsuitable for arteriovenous fistula.^1^ Unfortunately, up to 80% of AVG’s fail annually, leading to disruptions of dialysis schedules, reduced quality of life, and increased health care costs.^2,3^ The most common mechanism for AVG dysfunction is thrombosis, usually due to flow restriction from an underlying stenotic lesion.^4^

Thrombolysis with adjunctive thrombectomy and angioplasty ± stent is a minimally invasive treatment option for AVG thrombosis.^5^ This method facilitates disintegration and removal of thrombus and restores stenotic vascular segments to a functional state.^5^ Technical and immediate clinical success rates are high in most reported series, but long-term outcomes are unclear.^6,7^ We conducted a multicenter retrospective cohort study to analyze 5-year patency rates of AVG’s following thrombolysis.

## Methods

### Population

The research ethics board at the University Health Network, Toronto, Canada approved this study. All patients who underwent AVG thrombolysis between January 1, 2005 and December 31, 2015 at three academic hospitals including Toronto General Hospital, Mount Sinai Hospital, and Toronto Western Hospital were included. For patients who had multiple thrombolysis procedures, their first intervention was considered the index event. No patients had recorded thrombolysis procedures prior to 2005. Patients with primary or early AVG failure (never used for dialysis or failure within 3 months of creation) were also excluded.

### Pre-operative evaluation

All patients with AVG dysfunction were identified in the dialysis unit by a hemodialysis nurse and referred to a nephrologist, vascular surgeon, or interventional radiologist for further evaluation. Patients underwent clinical examination and ultrasound evaluation or fistulography to diagnose AVG thrombosis prior to intervention.

### Procedural protocol

Patients undergoing thrombolysis were informed about the benefits/risks of the procedure and provided informed consent. The procedure was performed in the interventional radiology suite under sedation and local anesthesia. Using ultrasound guidance, the AVG was accessed through needle puncture of the arterial and venous ends of the AVG. This is followed by wire, sheath, and catheter access. A fistulogram is performed through contrast injection into both the arterial and venous segments to determine the extent of thrombosis, including central vein involvement. Thrombolysis is then performed by injecting a mixture of 6mg IV tissue plasminogen activator (tPA) and 3000U IV heparin throughout the thrombosed segments. An over-the-wire Fogarty catheter and aspiration from the sheath sidearm is used for mechanical thrombectomy when appropriate. Stenotic segments are treated with adjunctive angioplasty ± stent. A completion angiogram is performed to confirm AVG patency, and the radiologist measured residual stenosis based on fimaging findings. Hemostasis is achieved using purse string sutures and Woggle technique.^8^ The patient is transferred to the recovery unit and monitored for at least 1 h prior to discharge.

### Data collection

Our study used data from prospectively maintained institutional nephrology and radiology databases. Baseline characteristics were recorded, including age, gender, race, comorbidities, AVG site, and time from AVG access creation to thrombolysis. The anatomic location of AVG stenosis were determined based on angiographic findings reported in procedural records.

### Study outcomes

The primary outcome was post-intervention (i.e. post-thrombolysis) primary patency (hereon “primary patency”), defined as AVG survival without re-intervention including angioplasty ± stent with/without re-thrombolysis. Secondary outcomes were post-intervention assisted primary patency (hereon “assisted primary patency”) and post-intervention cumulative patency (hereon “cumulative patency”), defined as AVG access survival until re-thrombosis requiring re-thrombolysis or abandonment, respectively. Technical success was defined as restoration of flow with <30% residual stenosis. These definitions are based on the Society of Vascular and Interventional Radiology Quality Improvement Guidelines^9^ and the American Society of Nephrology and US Food and Drug Administration Kidney Health Initiative.^10^

### Follow-up

After thrombolysis, patients are followed in the dialysis unit and referred to the vascular access clinic for further evaluation when there are signs of AVG access dysfunction as determined by a hemodialysis nurse. Data was collected until June 1, 2017.

### Statistical analysis

Continuous variables were reported as mean ± standard deviation (SD) or median (interquartile range; IQR) and categorial variables were reported as number and proportion. Primary, assisted primary, and cumulative patency rates were calculated using Kaplan–Meier survival analysis with patients censored for death, transplantation, change in renal replacement therapy modality, and loss to follow-up. Cox proportional hazards analysis was performed to determine associations between covariates and patency loss. Significance was set at *p* < 0.05. All analyses were performed in R (version 4.0.3) using survival version 3.2.7 and survminer version 0.4.8 packages.

## Results

Seventy-four patients underwent AVG thrombolysis during the study period with a median follow-up period of 21.4 (IQR 8.3–42.8) months. Thirteen patients were censored due to death (2), kidney transplant (6), and loss to follow-up (5). Average age was 58.6 years, and most were male (51.3%) and white (64.9%). Comorbidities including hypertension (82.4%) and diabetes (54.1%) were common. Most AVG configurations were either forearm loop (48.6%) or upper arm straight (36.5%). The mean time to thrombolysis was 17.9 (IQR 7.0–45.0) months (Table 1).

**Table 1.** Baseline patient characteristics.

	*N* = 74
Demographics	
Age (mean years ± SD)	58.6 ± 15.6
Female, no. (%)	36 (48.7%)
White, no. (%)	48 (64.9%)
Comorbidities	
Hypertension, no. (%)	61 (82.4%)
Diabetes, no. (%)	40 (54.1%)
Peripheral vascular disease, no. (%)	3 (4.1%)
Cerebrovascular disease, no. (%)	3 (4.1%)
AVG	
Forearm loop, no. (%)	36 (48.6%)
Upper arm straight, no. (%)	27 (36.5%)
Upper arm loop, no. (%)	8 (10.8%)
Forearm straight, no. (%)	3 (4.1%)
Time to thrombolysis (median months [IQR])	17.9 [7.0–45.0]

SD: standard deviation; IQR: interquartile range.

The most common anatomic location of AVG stenosis was within the graft (94.6%) followed by the venous anastomosis (51.4%), outflow vein (29.7%), and central vein (17.6%). Stenosis at either the arterial anastomosis (6.8%), or inflow artery (1.4%) were less common (Table 2).

**Table 2.** Anatomic locations of AVG stenosis.

	*N* = 74
Intra-graft, no. (%)	70 (94.6%)
Venous anastomosis, no. (%)	38 (51.4%)
Outflow vein, no. (%)	22 (29.7%)
Central vein, no. (%)	13 (17.6%)
Arterial anastomosis, no. (%)	5 (6.8%)
Inflow artery, no. (%)	1 (1.4%)

**Figure 1.**
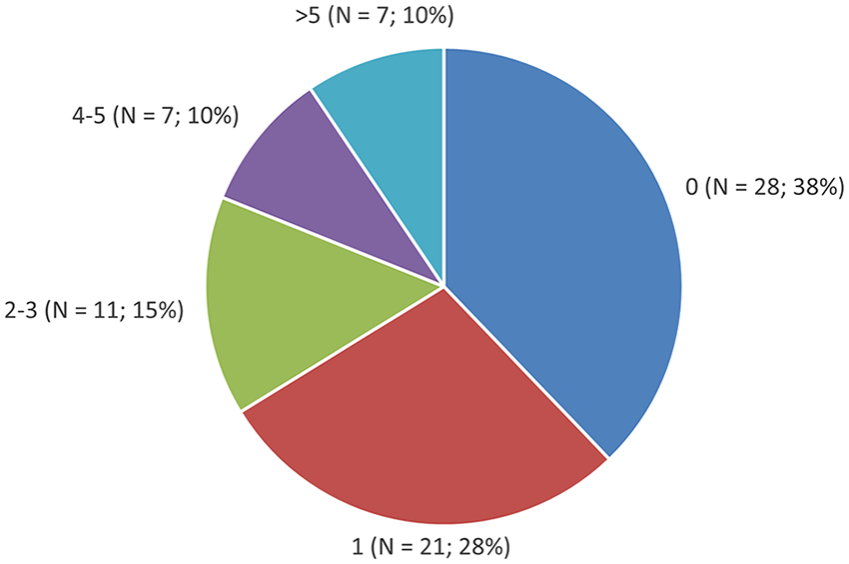
Number of re-interventions following AVG thrombolysis.

The technical success rate of thrombolysis was 96%. The majority of patients received adjunctive mechanical thrombectomy (93.2%) or angioplasty ± stent (82.4%) during their index procedure. Specifically, 21.6% of patients received a stent. There were 147 re-interventions in 46 patients, of which 98 were re-thrombolysis (mean re-intervention rate of 1.27/patient/year). Most patients had either 0 (38%) or 1 (28%) re-interventions, but a large number of patients had 2–3 (15%), 4–5 (10%), or > 5 (10%) re-interventions (Figure 1).

**Figure 2.**
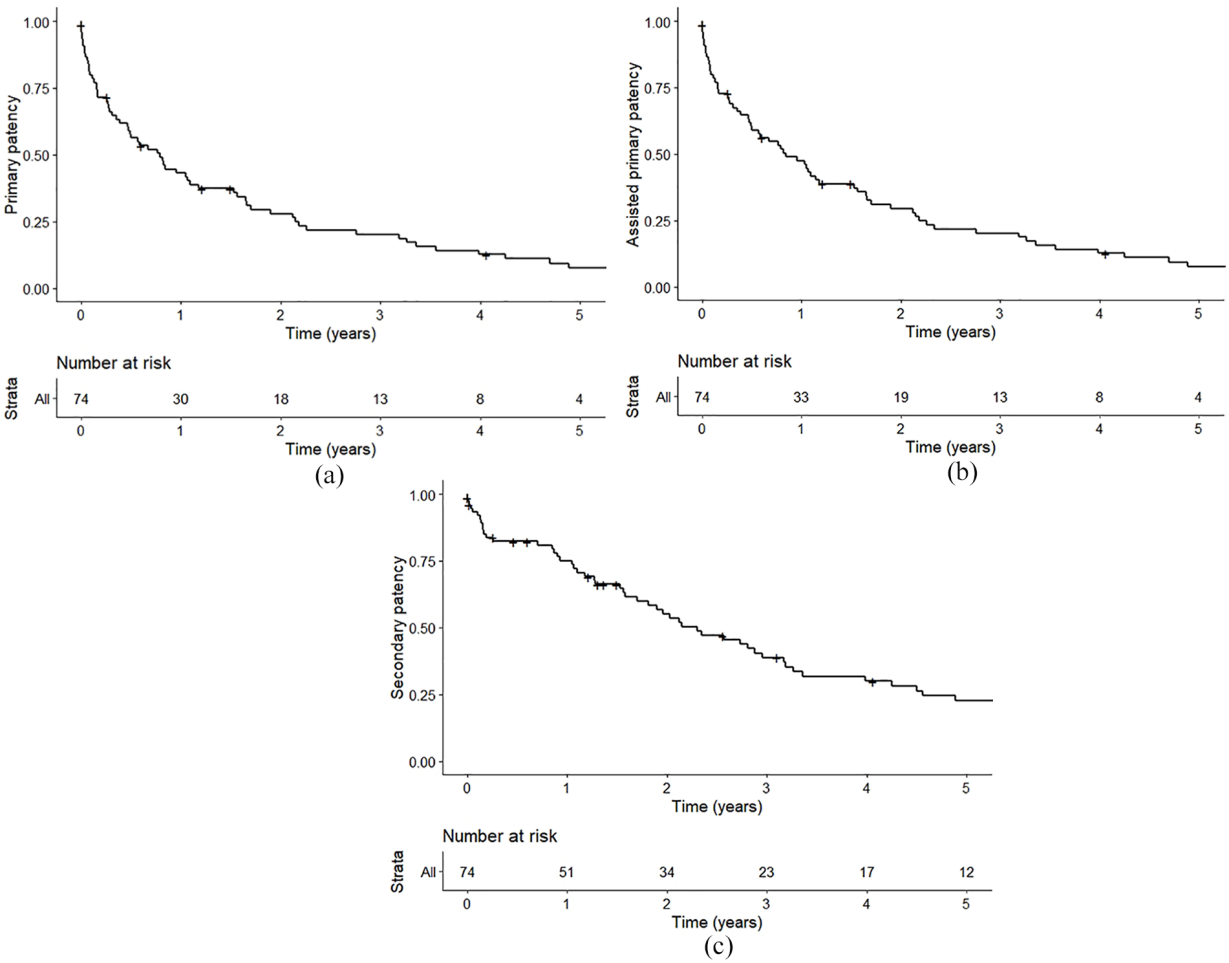
(a) Primary patency following AVG thrombolysis, (b) assisted primary patency following AVG thrombolysis, and (c) cumulative patency following AVG thrombolysis.

The primary patency rate following AVG thrombolysis at 1, 3, and 5 years were 43.2%, 20.2%, and 7.7% (Figure 2(a)). The assisted primary patency at 1, 3, and 5 years were 47.5%, 20.2%, and 7.7% (Figure 2(b)). The cumulative patency at 1, 3, and 5 years were 75.0%, 38.8%, and 22.6% (Figure 2(c)). These results are summarized in Table 3. Cox proportional hazards analysis demonstrated no associations between demographic, clinical, and procedural characteristics captured in our study and primary, assisted primary, or cumulative patency rates.

**Table 3.** Long-term AVG patency following thrombolysis.

	1 year	2 years	3 years	4 years	5 years
Primary patency	43.2%	27.9%	20.2%	12.7%	7.7%
Assisted primary patency	47.5%	29.4%	20.2%	12.7%	7.7%
Cumulative patency	75.0%	55.0%	38.8%	30.0%	22.6%

## Discussion

In our multicenter retrospective cohort study over a 10-year period, we demonstrated that despite a 96% technical success rate of AVG thrombolysis, long-term outcomes are poor, with a primary patency rate of 7.7% at 5 years. Although cumulative patency was better (22.6% at 5 years), this came at the cost of a significant number of additional procedures, with 20% of patients receiving four or more re-interventions. We did not find any associations between demographic, clinical, or procedural characteristics and patency in our population.

We demonstrated 1-year primary and cumulative patency rates of 43.2% and 75.0%, which are within range of the respective rates of 24%–70% and 44%–80% reported in the literature.^11,12^ We also surpassed the benchmark 1-year cumulative patency rate of 43% recommended by the Society of Interventional Radiology Quality Improvement Guidelines.^13^ However, our longer-term results demonstrate a significant decline in patency up to 5 years.

Yilmazsoy and Ozyer (2019) analyzed long-term outcomes in patients undergoing thrombolysis for arteriovenous fistulas (AVF) and AVG’s. Their subgroup analysis of AVG’s demonstrated similarly poor patency rates, with primary patency at 3 years of 0% and 5-year assisted primary and cumulative patency rates of 1% and 48%, respectively. In their study, AVF’s had significantly better outcomes, with primary patency of 45% at 3 years and assisted primary patency of 30% at 5 years.^14^ We confirmed poor long-term outcomes in our study dedicated to AVG’s.

Notably, a significant proportion of our study population received adjunctive mechanical thrombectomy (93.2%) and angioplasty ± stent (82.4%). This is similar to the adjunctive procedure rate of 90.8% reported by Yilmazsoy and Ozyer.^14^ Therefore, despite aggressive use of additional procedures to salvage AVG’s, long-term patency remains poor. In our cohort, 21.6% of patients received a stent, which was higher than the stent rate of 14.5% in the study by Yilmazsoy and Ozyer (2019). The low rate of stenting was likely due to the theoretical risk of neointimal hyperplasia and re-thrombosis. However, with improvements in stent technology, recent studies have demonstrated positive results with nitinol^15^ and covered stents^16,17^ for treatment of AVG stenosis. Future studies could assess the impact of stenting on long-term AVG patency following thrombolysis.

Several studies have identified factors associated with poor long-term patency following endovascular intervention for dysfunctional AV accesses. Huber et al. (2003) conducted a systematic review demonstrating that dysfunctional upper arm AVF’s have higher patency rates following endovascular intervention than forearm AVF’s.^18^ Our study did not find an association between AVG site and long-term patency, which may be because of poor AVG outcomes at any anatomic location. Others have reported that mechanical thrombectomy shortens procedural time, but does not improve patency rates.^19,20^ We similarly demonstrated no association between adjunctive mechanical thrombectomy and long-term patency.

In our study, the most common reason for re-intervention was re-thrombosis. This suggests that following the first thrombotic event, AVG’s are prone to further thrombosis. There may be several reasons for this. First, angioplasty ± stent of underlying stenotic lesions increases the risk of neointimal hyperplasia and future thrombosis.^21^ Second, underlying conditions contributing to elevated thrombotic risk are not addressed by thrombolysis, such as low cardiac output from congestive heart failure, hypovolemia from dehydration, or over ultrafiltration/hypotension during hemodialysis.^22^ Finally, patients with end stage kidney disease are in a chronic inflammatory and prothrombotic state, increasing their risk of AV access thrombosis.^23^ Together, these factors may explain the high initial technical success rate but poor long-term outcomes following AVG thrombolysis.

Anticoagulation has been investigated as a method to reduce the risk of AVG thrombosis. Crowther et al. (2002) conducted a randomized controlled trial comparing warfarin to placebo in patients with polytetrafluoroethylene (PTFE) AVG’s.^24^ The trial was prematurely terminated due to a significant increase in major bleeding with no difference in graft survival.^24^ Therefore, anticoagulation is not routinely prescribed to prevent thrombosis in patients with AVG. Anticoagulation for the sole purposes of maintaining vascular access patency is also not recommended in the new KDOQI vascular access guidelines 2019, due to lack of benefit and suggestion of harm.^25^ Future studies should investigate new strategies to improve AVG patency in patients who have undergone AVG thrombolysis.

Some authors have argued for surgical thrombectomy as first-line treatment for AVG thrombosis.^26^ Green et al.^26^ conducted a meta-analysis demonstrating higher patency and lower technical failure rates for surgery compared to endovascular therapy. However, this study was conducted 2 decades ago and endovascular therapy has developed significantly. Furthermore, surgical thrombectomy carries the risks of open surgery, including wound complications, longer procedural time, and prolonged recovery. The success of surgical versus endovascular treatment as first line therapy may depend on the location of the lesion. It would make intuitive sense that peri-anastomotic lesions repaired by open surgery might have superior patency while lesions within the body of the AV access may respond equally well or better with endovascular intervention. Therefore, contemporary comparisons between surgical and endovascular treatment options for AVG thrombosis are needed. We look forward to the results of the Cochrane systematic review by Fonseca and colleagues (2019), which will provide important insight into the comparative effectiveness of surgical and endovascular interventions for AVG thrombosis in the modern era.^27^

Our study has several limitations. First, this is a retrospective analysis of our experience in a large academic health network. Second, thrombolysis was only performed on upper extremity AVG’s during our recruitment period and future studies should investigate whether similar outcomes exist for lower extremity AVG’s. Third, our study was not designed to detect associations between covariates and patency rates, and therefore, our inability to identify predictors for patency loss may be due to an underpowered sample size. Fourth, AVG indication was not recorded; however, we expect that the majority were for end stage renal disease. More specifically, we did not record whether an AVG was placed as the first access for hemodialysis or to replace an existing AVF/AVG/central line that has become thrombosed or infected. Future studies could assess whether AVG indication affects long-term patency following thrombolysis.

## Conclusions

Thrombolysis for AVG dysfunction yields a high initial technical success rate, however, long-term patency is poor. Twenty percent of patients undergo four or more re-interventions following index thrombolysis, which may contribute to decreased quality of life and increased health care costs. Further investigation into risk factors for re-thrombosis are needed to identify patients who will benefit from AVG thrombolysis in the long-term.
